# Knowledge structure and hotspots research of glioma immunotherapy: a bibliometric analysis

**DOI:** 10.3389/fonc.2023.1229905

**Published:** 2023-08-21

**Authors:** Yexin Yuan, Yue Su, Yingxi Wu, Yafei Xue, Yunze Zhang, Yangyang Zhang, Min Zheng, Ting Chang, Yan Qu, Tianzhi Zhao

**Affiliations:** ^1^ Department of Neurosurgery, Tangdu Hospital, Air Force Medical University, Xi’an, Shaanxi, China; ^2^ Department of Neurology, Tangdu Hospital, Air Force Medical University, Xi’an, Shaanxi, China

**Keywords:** glioma, immunotherapy, bibliometric, Citespace, VOSviewer, ICIs

## Abstract

**Background:**

Glioma is the most common primary brain tumor. Traditional treatments for glioma include surgical resection, radiotherapy, chemotherapy, and bevacizumab therapy, but their efficacies are limited. Immunotherapy provides a new direction for glioma treatment. This study aimed to summarize the knowledge structure and research hotspots of glioma immunotherapy through a bibliometric analysis.

**Method:**

Publications pertaining to glioma immunotherapy published during the period from 1st January 1990 to 27th March 2023 were downloaded from the Web of Science Core Collection (WoSCC). Bibliometric analysis and visualization were performed using the CiteSpace, VOSviewer, Online Analysis Platform of Literature Metrology, and R software. The hotspots and prospects of glioma immunotherapy research were illustrated via analyzing the countries, institutions, journals, authors, citations and keywords of eligible publications.

**Results:**

A total of 1,929 publications pertaining to glioma immunotherapy in 502 journals were identified as of 27th March 2023, involving 9,505 authors from 1,988 institutions in 62 countries. Among them were 1,285 articles and 644 reviews. Most of publications were produced by the United States. *JOURNAL OF NEURO-ONCOLOGY* published the majority of publications pertaining to glioma immunotherapy. Among the authors, Lim M contributed the largest number of publications. Through analyzing keyword bursts and co-cited references, immune-checkpoint inhibitors (ICIs) were identified as the research focus and hotspot.

**Conclusion:**

Using a bibliometric analysis, this study provided the knowledge structure and research hotspots in glioma immunotherapy research during the past 33 years, with ICIs staying in the current and future hotspot. Our findings may direct the research of glioma immunotherapy in the future.

## 1 Introduction

Glioma, the most common primary brain tumor, usually originates from glial cells or precursor cells and appears in many forms, such as astrocytoma, oligodendroglioma, and ventricular meningioma (1, 2). According to the revised 2016 WHO classification of CNS tumors, glioma is classified into four grades. Adult glioma is classified into three main categories based on the absence or presence of mutations in IDH, or 1p and 19q (1p/19q) chromosomal co-deletion: IDH-mutant, 1p/19q-co-deleted tumors which are predominantly oligodendrocytic; IDH-mutant, non-1p/19q-co-deleted tumors which usually have an astrocytic form; and IDH-wild-type tumors which are dominated by glioblastoma (GBM) (WHO grade IV) (3). GBM is the most malignant glioma. In the US, the total annual incidence of glioma is approximately 6 cases per 100,000 people, with half made up by GBM cases and the majority by males (4). Traditional treatments for glioma include surgical resection, radiotherapy, chemotherapy, and bevacizumab therapy, but their efficacies are limited. Owing to the diffuse growth of glioma, surgery cannot remove the entire tumor, thus resulting in a high recurrence rate (5). In 2005, Stupp R’s team found that temozolomide combined with radiotherapy shows significant benefit in the treatment of patients with GBM (6). Friedman HS’s team have confirmed the antitumor activity of bevacizumab (a synthetic drug targeting the monoclonal antibody vascular endothelial growth factor [VEGF]) and bevacizumab in combination with irinotecan (7). However, no evidence is available to support that conventional therapeutic interventions can significantly prolong the survival of patients with tumor relapse (2, 8, 9). In addition, the blood-brain barrier restricts the distribution of drugs, the aggressive growth of tumors fails the local treatment, and the immunosuppression and high toxicity limit the use of traditional drugs, all bringing a poor prognosis to patients with glioma. Therefore, there is an urgent need to develop new therapies.

With the discovery of functional lymphatic vessels within the dural sinuses (10), more evidence suggests the existence of active immune surveillance in the central nervous system (CNS) (9). In contrast, microglia/macrophages (GIMs) function as major immune cells, but they play a negative role in the glioma microenvironment (11). Glioma cells release some immunosuppressive factors, such as transforming growth factor-β (TGF-β), thus allowing for immune escape (12). New data indicate that the immunosuppressive environment of glioma is multifactorial. However, immunotherapy has shown to modify this environment by inhibiting some cytokines or suppressing tumor cells, thus exerting favorable anti-tumor effects. Therefore, immunotherapy may open a new avenue for the treatment of glioma.

Research on glioma immunotherapy is extensive, involving basic and clinical studies. Immunotherapy can be accomplished in many approaches, including vaccine therapy, viral therapy, CAR-T therapy, and immune checkpoint inhibitors (ICIs). Related studies have been well reviewed from various perspectives. However, there have been no analyses encompassing the publication volume, influential countries/regions, institutions, authors, global collaborations, knowledge structure and research hotspots.

Bibliometric analysis is a common method to qualitatively and quantitatively analyze the characteristics of publications, such as countries, institutions, and research collaboration (13–15). Through this tool, the knowledge structure and research hotspots of a certain field can be sorted out, thereafter providing references for future research (16, 17). To our knowledge, bibliometric analysis has never been employed to evaluate the studies about glioma immunotherapy. Based on publications screened out of the Web of Science, this study for the first time summarized the knowledge structure and research hotspots of glioma immunotherapy, using bibliometric analysis combined with other statistical tools of CiteSpace, VOSviewer, R software, and Online Analysis Platform of Literature Metrology.

## 2 Materials and methods

### 2.1 Data collection

**Figure 1:**
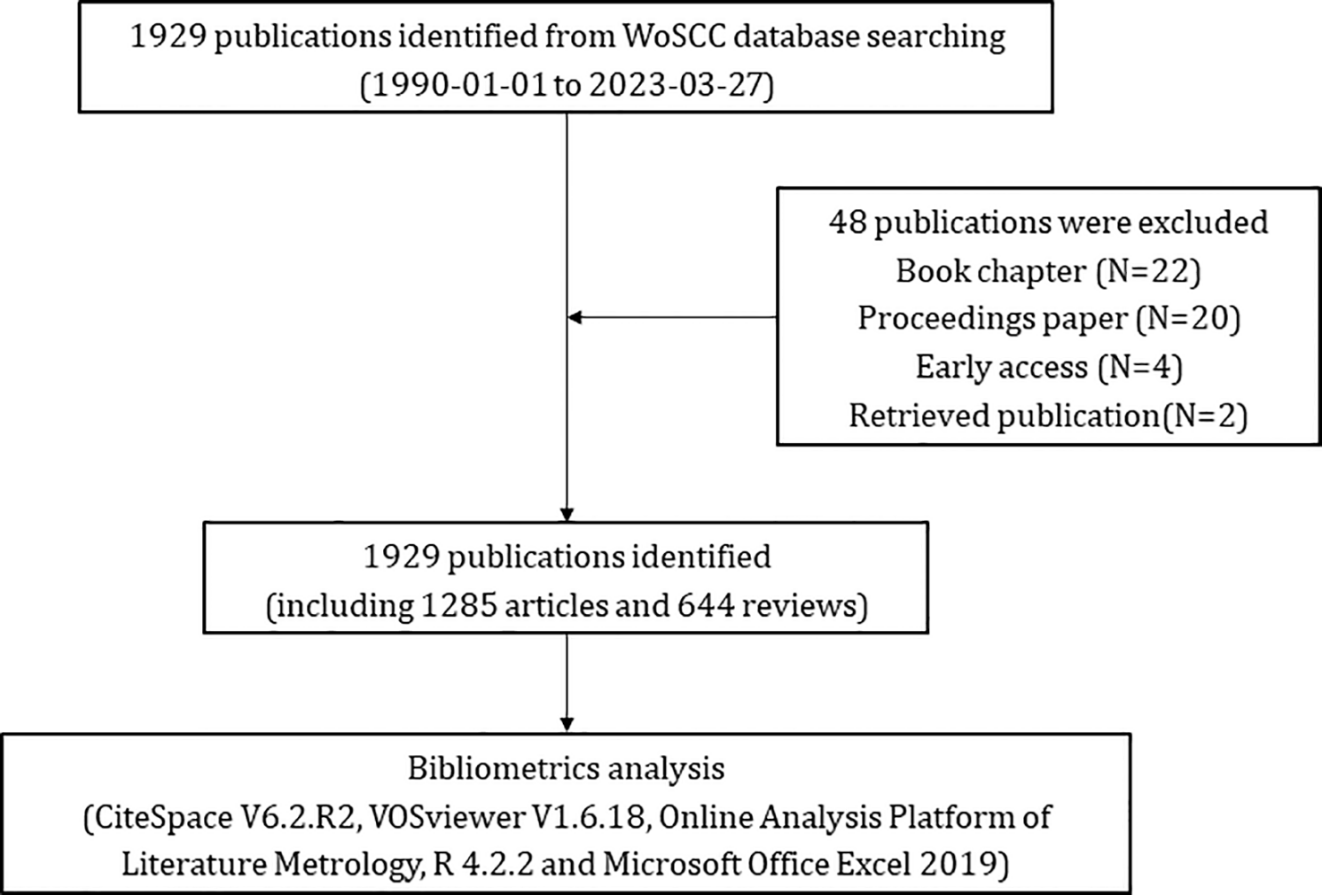
Flowchart of screening eligible publications pertaining to glioma immunotherapy.

The Web of Science Core Collection (WoSCC) is one big and comprehensive database for scientific research (18), with more than 12 000 high-quality journals. Also, previous studies have indicated that WoSCC is the most appropriate database for bibliometric analysis (19). According to the literature related to glioma, we determined the common keywords about glioma, and then we preliminarily formulated the search terms related to “glioma”. At the same time, by reading the original articles and reviews of glioma immunotherapy, we have a preliminary understanding of the mainstream immunotherapy methods for glioma, which have been included in the relevant key words of “immunotherapy”. Taking the intersection of the two, we get our search formula. Publications pertaining to glioma immunotherapy were searched from WoSCC with the following queries: #1: TI=(“glioma”) OR AK=(“glioma”) OR TI=(“malignant glioma”) OR AK=(“malignant glioma”) OR TI=(“high grade glioma”) OR AK=(“high grade glioma”) OR TI=(“glioblastoma”) OR AK=(“glioblastoma”); #2: TI=(“immunotherapy*”) OR AK=(“immunotherapy*”) OR TI=(“immune NEAR/2 therap*”) OR AK=(“immune NEAR/2 therap*”) OR TI=(checkpoint inhibitor) OR AK=(checkpoint inhibitor) OR TI=(PD-1) OR AK=(PD-1) OR TI=(PD-L1) OR AK=(PD-L1) OR TI=(nivolumab) OR AK=(nivolumab) OR TI=(pembrolizumab) OR AK=(pembrolizumab) OR TI=(CAR-T) OR AK=(CAR-T) OR TI=(chimeric antigen receptor T-cell therapy) OR AK=(chimeric antigen receptor T-cell therapy) OR TI=(dendritic cell vaccines) OR AK=(dendritic cell vaccines) OR TI=(viral therapy) OR AK=(viral therapy); the ultimate dataset: #1 AND #2. Only English-written articles or reviews published during the period from 1st January 1990 to 27th March 2023 were chosen. A truncation symbol “*” was used and the use of truncation searches improved recall and prevented missing inspection. The articles with research content related to the theme of “glioma immunotherapy” were included by reading the titles, abstracts and keywords of the detected articles. Articles with incomplete research information, book chapters, proceedings papers, early accesses, retrieved publications and duplicate articles were excluded. Eventually, after excluding 22 book chapters, 20 proceedings papers, 4 early accesses, 2 retrieved publications, a total of 1,929 eligible publications were included (**Figure 1**). All data used in this work were downloaded from a public database; therefore, no ethics approval or informed consent was required. The above processes were carried out under the guidance of glioma experts and bibliometrics experts.

### 2.2 Data analysis and visualization

The publications were downloaded and their data were put into Microsoft Office Excel 2019, analyzed and visualized using CiteSpace 6.2.R2, VOSviewer 1.6.18, Online Analysis Platform of Literature Metrology (https://bibliometric.com/) and R 4.2.2 (20–22).

Tables were depicted using Microsoft Office Excel. The Online Analysis Platform of Literature Metrology was used to visualize networks of collaborations between countries/regions and trends in the volume of publications over time. R software was used to visualize the collaborative relationships between countries, authors, and institutions, and to analyze references.

VOSviewer is a widely-used software in bibliometric analysis developed by van Eck and Waltman. It is powerful in building and visualizing bibliometric networks, such as the co-authorship, co-occurrence and co-citation networks of counties, organizations, journals, authors and keywords (23, 24). It was used in the present study to visualize co-authorship between authors, institutions and countries, and the co-occurrence of keywords. Co-authorship analysis is a measure to establish similar relationships among items through the number of coauthored documents, and co-occurrence analysis is to build a network that represents the relationships between items according to the quantity of publications occurring together. Co-citation refers to that two documents were cited simultaneously in the bibliography of the third document (19, 23).

CiteSpace is another information visualization software for bibliometric analysis founded by Professor Chaomei Chen (25). This software can be used to find the key points, especially intellectual pivotal points, or turning points in the research history of a certain field, and predict the research trend through data mining. Additionally, this software is unique in that it can be used to build a dual map of journals, which is generated in the context of 10 000 journals indexed in WoSCC (24). In this study, it was used to (1) analyze the countries, institutions and authors involved in the study; (2) construct dual-map of journals involved; (3) demonstrate reference clustering and references/keywords burst. In CiteSpace, the results are visualized as nodes and lines, and for these nodes, the centrality score is calculated to measure the relative importance of a node in a network. When a node has a centrality score greater than or equal to 0.1, it is usually considered a relatively important hub node (19, 25). In co-cited reference clustering, CiteSpace adopts several metrics to measure the outcome clusters, which include modularity and silhouette. The modularity measures the significance of the divided modules. While the modularity value is greater than 0.3, the clustering structure is usually considered as significant. The silhouette is an indicator used to measure the consistency of a cluster. A cluster is considered reasonable or convincing when its values are greater than 0.5 or 0.7, respectively (19, 26).

## 3 Results

### 3.1 Annual growth trend of publications and citations

**Figure 2:**
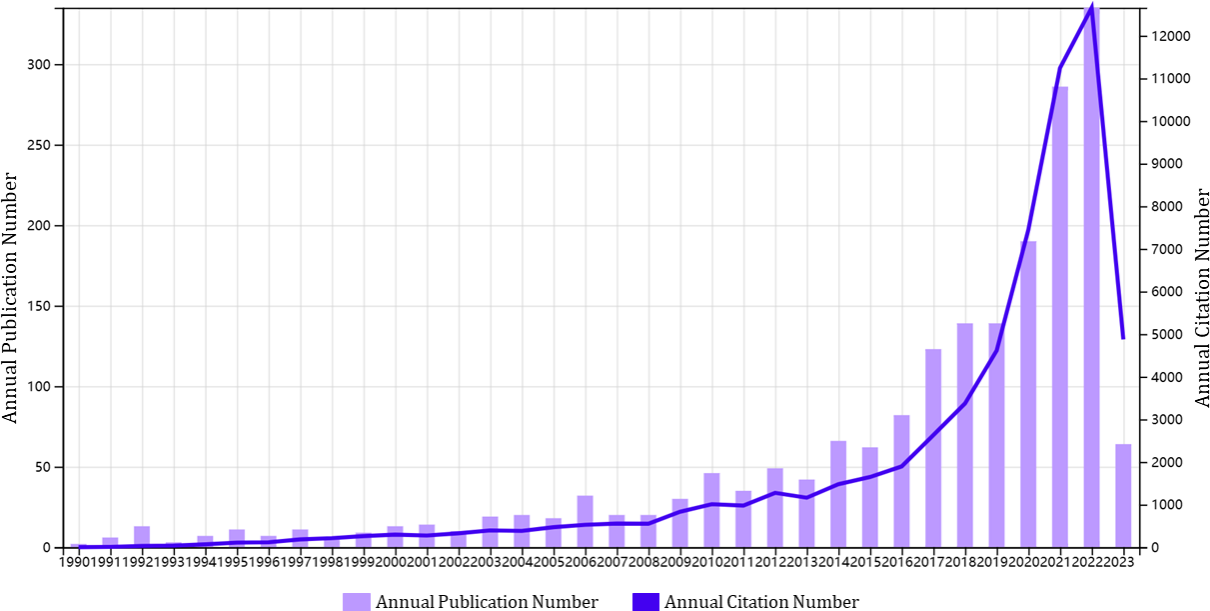
A trend in the annual publications on glioma immunotherapy. The purple column represents annual publication number, and the blue line represents the development trend of annual citation number.

From 1st January 1990 to 27th March 2023, a total of 1,929 publications pertaining to glioma immunotherapy were identified, including 1,285 articles and 644 reviews (**Figure 1**). All of them had been cited for 61,875 times, and 47,878 times after removing self-citations. The mean citation and H-index of a publication were 32.08 times and 109, respectively. Until 2005, just less than 20 articles were published in this field each year (**Figure 2**). However, in the following decade (except for a few short periods showing a decrease in publication volume), articles related to glioma immunotherapy flourished gradually. Starting from 2006, the annual number of articles published kept increasing, with over 30 articles in each of the 8 years. Starting from 2016, the number of articles in this field grew rapidly. Except for the number of publications in 2019 remained the same as that in the previous year, the number of articles published in all other years had continuously increased, so did the number of citations. The number of articles published in 2023 (as of March) had reached 30, and is predicted to explode in the future.

### 3.2 Analysis of countries/regions and institutions

**Figure 3:**
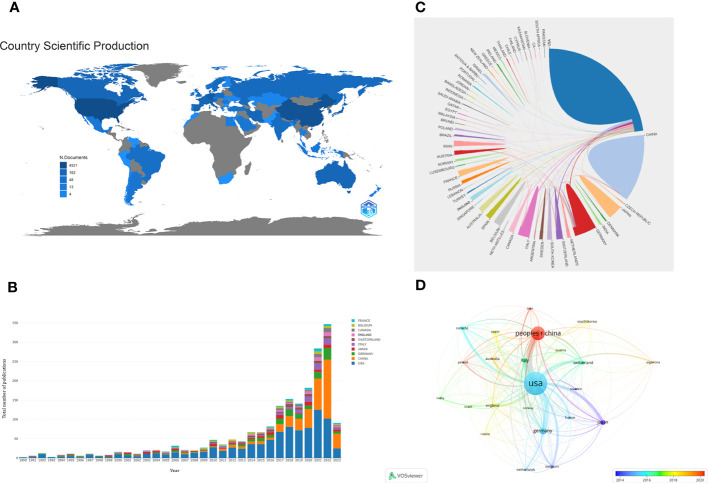
**(A)** A geographic distribution map displaying the global contributions to the research of glioma immunotherapy. The darker the color (blue), the more publications published in this country, and gray represents no publication. **(B)** The annual numbers of publications from the top 10 countries/regions between 1990 and 2023. Each color represents a country. **(C)** A network map displaying the collaboration between countries/regions. Each country corresponds to an arc area of different colors, and the size of the area represents the number of publications. The line between arcs represents the cooperative relationship between countries. The thickness of the line represents the intensity of cooperation. **(D)** A co-authorship network between countries/regions based on time by VOSviewer. Each node represents a country/region, and the connection lines between nodes represent a cooperative relationship between the two. The thickness of the line represents the intensity of cooperation. The color of each node corresponds to the color on the timeline and represents the time when cooperation occurs with other nodes.

A total of 62 countries participated in the research of glioma immunotherapy. **Table 1** presents the top 10 countries/regions with the largest numbers of articles published, and **Figure 3A** presents a map of the contributions from different countries. The top country was the United States (n=918, 47.589%), followed by China (n=429, 22.240%), Germany (n=165, 8.554%), Japan (n=104, 5.391%), and Italy (n=101, 5.236%), while the rest countries/regions had released less than 60 publications. Among them, the number of citations of published articles from the United States was 39,991, far exceeding the 7,460 from China, in the second place, and the 7,296 from Germany, in the third place. H-index indicated that each of at least H publications was cited for at least H times (27). A high H-index is parallel to a high academic influence of a country. The United States ranked first with a score of 97, far above other countries. Centrality score of a single country indicates the position of a country in this scientific field. The United States ranked first with a score of 0.64, followed by Germany (centrality=0.23), China (centrality=0.11), France (centrality=0.11); the other countries in the top ten achieved a centrality less than 0.1. As shown in **Figure 3B**, the US had a relatively stable contribution, which was manifested by its stable number of articles published in this field every year; China, the second contributor, published a large number of articles in the last few years, especially in 2021 and 2022, which could be confirmed by the trend chart of China’s publications over the years (**Supplementary Figure 1**). It is also worth mentioning that China ranked second in the numbers of publications (429). China published articles in a number almost one-half of that in the US (**Table 1**), but its total number of citations was just one-fifth of that in the US (7,460), and its average number of citations per article ranked the bottom in the top ten (17.43). **Figures 3C**, **D** show the cooperation patterns between different countries/regions. International cooperation was still relatively frequent, mostly occurring between European countries, East Asian countries, and American countries. Among them, cooperation with the US or cooperation between the US and China was the most frequent. In **Figure 3D**, the colors corresponding to the nodes and timeline represent the time of cooperation between the two. Around 2015, the US was the most active country in this research field, cooperating with many countries. However, around 2020, China became the most active country, and in recent years, most countries have enhanced their cooperation with China.

**Table 1:** Top 10 countries/regions contributing to the research on glioma immunotherapy.

Rank	Country	Number of publications	Number of citations	Citations of per article	H-Index	Centrality
1	USA	918	39991	43.56	97	0.64
2	CHINA	429	7460	17.43	45	0.11
3	GERMANY	165	7296	44.22	47	0.23
4	JAPAN	104	3460	33.27	34	0.02
5	ITALY	101	2984	29.54	31	0.05
6	SWITZERLAND	59	4811	81.54	31	0.02
7	CANADA	53	2261	42.66	24	0.04
8	BELGIUM	49	1954	39.88	24	0.02
9	FRANCE	49	1702	34.73	23	0.11
10	ENGLAND	47	2033	43.26	19	0.07

**Figure 4:**
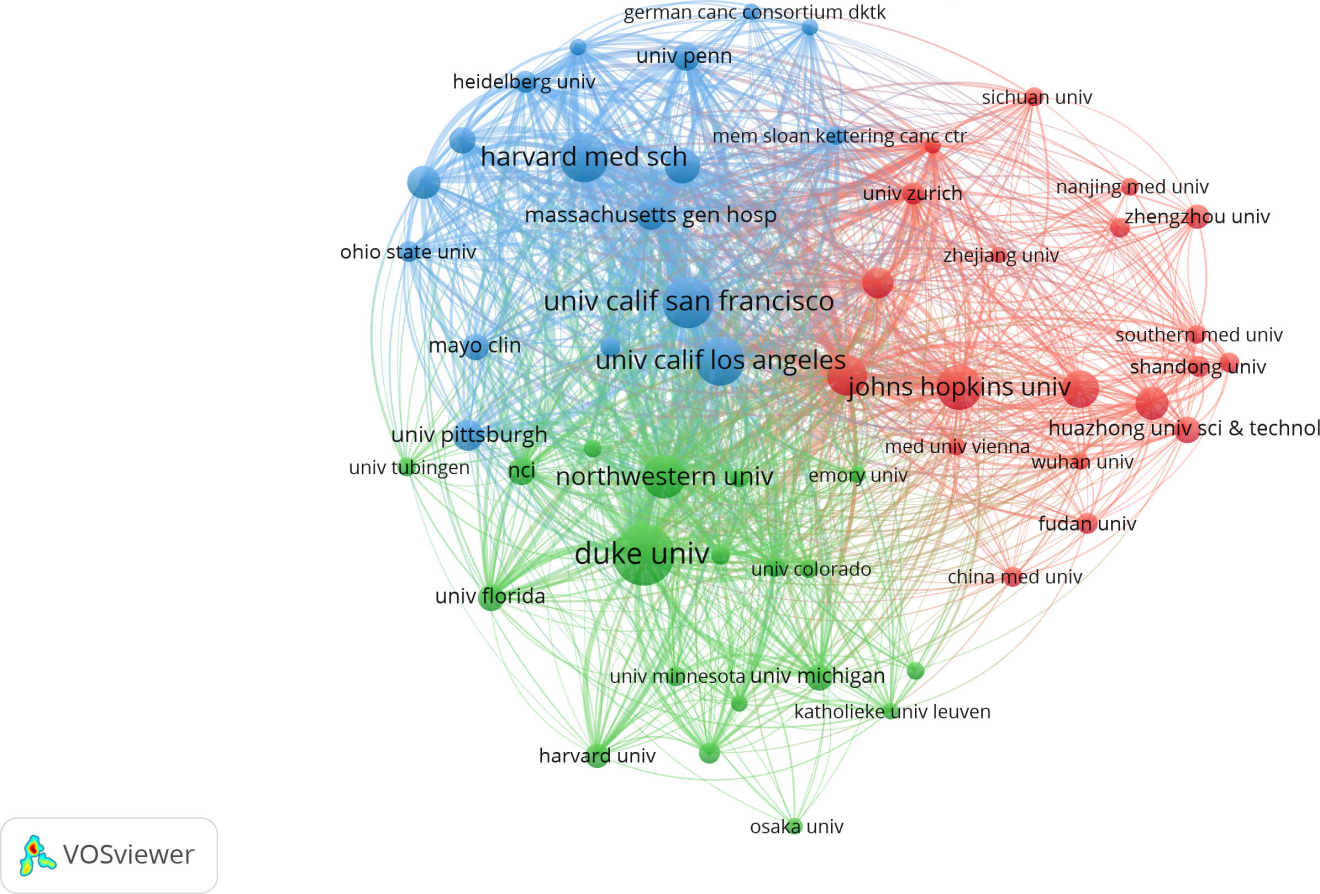
The network of collaboration between institutions contributing to the research on glioma immunotherapy by VOSviewer. Each node represents an institution. The size of the node represents the number of publications issued by the institution. Nodes with the same color represent that they belong to the same cluster, indicating that their research are similar.

**Table 2** shows the top 10 institutions with the highest total numbers of publications, with Harvard University (n=118) leading in the top five, followed by Duke University (n=82), Harvard Medical School (n=75), Johns Hopkins University (n=69) and University of California San Francisco (n=65). In terms of citations, however, the top five changed to Harvard University (7261), Duke University (4985), UTMD Anderson Cancer Center (4414), University of California San Francisco (4327) and Johns Hopkins University (4001). Harvard University, Duke University, Johns Hopkins University and the University of California San Francisco ranked the top five in terms of either total publications or total citations. Notably, UTMD Anderson Cancer Center, which ranked only 7th in total publications with 55, rose to the third place in the total citations with 4414. In addition, among the top ten institutions, all but University of California Los Angeles (centrality 0.1) had centrality values less than 0.1. The different colors in **Figure 4** represent different clusters, and the major institutions are divided into four clusters. The research content in each cluster was closer and more collaborative, which could also be confirmed by the graph drawn by R Software (**Supplementary Figure 2**). Cooperation among major institutions was relatively regional. For example, US institutions were more likely to cooperate within the US, while major Chinese institutions tended to work together. But this did not mean the lack of international cooperation (**Figure 4**; **Supplementary Figure 2**).

**Table 2:** Top 10 institutions contributing to the research on glioma immunotherapy.

Rank	Institution	Number of publications	Number of citations	H-index	Centrality
1	HARVARD UNIVERSITY	118	7261	42	0.07
2	DUKE UNIVERSITY	82	4985	37	0.04
3	HARVARD MEDICAL SCHOOL	75	3426	31	0.01
4	JOHNS HOPKINS UNIVERSITY	69	4001	31	0.04
5	UNIVERSITY OF CALIFORNIA SAN FRANCISCO	65	4327	28	0.02
6	UNIVERSITY OF CALIFORNIA LOS ANGELES	61	3571	30	0.10
7	UTMD ANDERSON CANCER CENTER	55	4414	31	0.08
8	HELMHOLTZ ASSOCIATION	53	2798	23	0.02
9	NORTHWESTERN UNIVERSITY	51	2484	25	0.03
10	GERMAN CANCER RESEARCH CENTER DKFZ	50	2668	21	0.02

### 3.3 Visualization of journals and co-cited journals

Of the 1929 articles published in 502 journals, the largest proportion (100, 5.184% of the total) was accepted by *JOURNAL OF NEURO-ONCOLOGY* (4.506, Q2) (**Table 3**), followed by *FRONTIERS IN IMMUNOLOGY* (8.787, Q1), *FRONTIERS IN ONCOLOGY* (5.738, Q2), *NEURO ONCOLOGY* (13.029, Q1), and *CANCERS* (6.575, Q1). Among the top 10 articles, nine journals had an impact factor (IF) of more than 5.0, and eight were in the Q1 JCR division, indicating that most of the articles had been published in journals of high quality and influence.

**Table 3:** Top 10 journals publishing the most articles about glioma immunotherapy and top 10 journals with the most co-cited publications pertaining to glioma immunotherapy.

Rank	Journal	Counts	IF and JCR division (2021)	Rank	Journal	Total number of citations	IF and JCR division (2021)
1	JOURNAL OF NEURO-ONCOLOGY	100	4.506, Q2	1	CLINICAL CANCER RESEARCH	1475	13.801, Q1
2	FRONTIERS IN IMMUNOLOGY	99	8.787, Q1	2	CANCER RESEARCH	1446	13.312, Q1
3	FRONTIERS IN ONCOLOGY	80	5.738, Q2	3	NEURO-ONCOLOGY	1382	13.029, Q1
4	NEURO-ONCOLOGY	76	13.029, Q1	4	NEW ENGLAND JOURNAL OF MEDICINE	1296	176.082, Q1
5	CANCERS	66	6.575, Q1	5	JOURNAL OF NEURO-ONCOLOGY	1162	4.506, Q2
6	CANCERS IMMUNOLOGY IMMUNOTHERAPY	57	6.63, Q1	6	NATURE	1100	69.504, Q1
7	ONCOIMMUNOLOGY	47	7.723, Q1	7	JOURNAL OF CLINICAL ONCOLOGY	1100	50.739, Q1
8	INTERNATIONAL JOURNAL OF MOLECULAR SCIENCES	34	6.208, Q1	8	PNAS	1087	12.779, Q1
9	CLINICAL CANCER RESEARCH	26	13.801, Q1	9	NATURE MEDICINE	1028	87.244, Q1
10	JOURNAL FOR IMMUNOTHERAPY OF CANCER	26	12.485, Q1	10	SCIENCE	940	63.832, Q1

While in the analysis of co-cited journals (**Table 3**), *CLINICAL CANCER RESEARCH* (13.801, Q1) had the highest number of co-citations, followed by *CANCER RESEARCH* (13.312, Q1), *NEURO ONCOLOGY* (13.029, Q1), *NEW ENGLAND JOURNAL OF MEDICINE* (176.082, Q1) and *JOURNAL OF NEURO-ONCOLOGY* (4.506, Q2). Among the top 10 journals cited, nine had an IF exceeding 10 and were in the Q1 JCR division. Among them were also famous journals, such as *NEW ENGLAND JOURNAL OF MEDICINE* (176.082, Q1), *NATURE* (69.504, Q1), *JOURNAL OF CLIMATIC ONCOLOGY* (50.739, Q1), *NATURE MEDICINE* (87.244, Q1), and *SCIENCE* (63.832, Q1).

**Figure 5:**
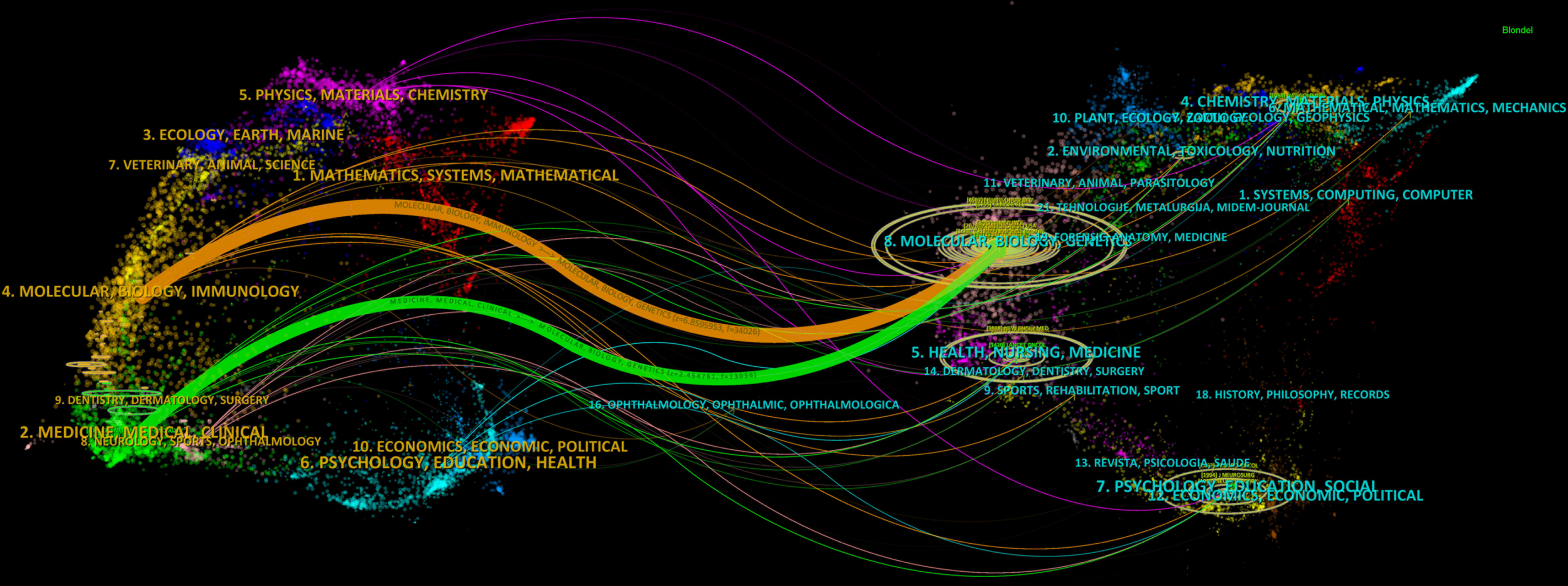
Dual-map overlay displaying trajectories between citing and cited journals. On the left is the citing literature; on the right is the cited literature, and the connection line between the two shows the citation pathway. The horizontal and vertical axes of the oval, respectively, represent the number of authors and publications, the length of which is positively correlated with the number.

A dual-map overlay displays the distribution of journals in different disciplines, and the trajectories between the cited and citing publications (28, 29). The distributions of citing journals (left) and cited journals (right) in each field are shown in **Figure 5**, in which the connection lines represent the trajectories through which the citing publications cited the cited publications. The horizontal and vertical axes of the oval, respectively, represent the number of authors and publications, the length of which is positively correlated with the number (28). Dual map contained three main pathways, showing that the articles in glioma immunotherapy mainly cited the research outcomes in Molecular Medicine, Biology, and Genetics, and were published in two different fields: Molecular Medicine, Biology, Immunology; and Medicine, Medical, Surgery.

### 3.4 Analysis of authors

**Figure 6:**
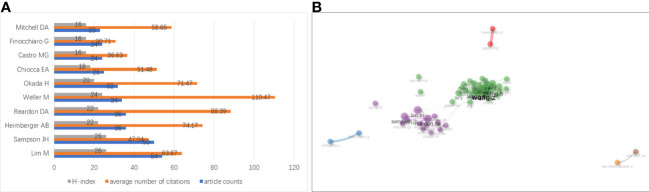
**(A)** Top 10 authors having published the most articles about glioma immunotherapy. The gray, orange and blue columns represent the H-index of each author, the average number of citations of each author and the number of papers published by each author, respectively. **(B)** A network analysis of collaborations between authors contributing to the research on glioma immunotherapy. Each node represents an author. The thickness of the line between two nodes represents the intensity of cooperation. Nodes with the same color represent that they belong to the same cluster, indicating that their research are similar.

The top 10 productive authors in the research on glioma immunotherapy are listed in **Figure 6A**. Lim M was the most productive, with 54 publications and 3449 citations. From the perspective of H-index, the gaps between the top 10 authors were not significant; but in terms of citation, Weller M left a significant gap in front of others. His articles had been cited for the largest times (3756 times), with each of his articles cited by 110.47 times averagely. In addition, as shown in **Figure 6B**, the collaborative relationships between authors indicated a lack of collaboration at the global level. The authors were more likely to collaborate in their own cluster, and the collaboration between different clusters remained to be expanded. This phenomenon is also visualized in **Supplementary Figure 3** drawn by VOSviewer. The nodes represent the clusters of authors, and there are fewer connections between clusters.

### 3.5 Analysis of references with citation clustering and bursts

**Table 4** shows the details of the top 10 most cited articles. The most cited article was produced by BROWN CE et al., published in *NEW ENGLAND JOURNAL OF MEDICINE* in 2016, and titled “Regression of Glioblastoma after Chimeric Antigen Receptor T-Cell Therapy” (30). In a clinical study conducted by their team, they administered CAR-T therapy targeting IL-13Ra2 to a patient with recurrent GBM, and observed a reduction in the size of all lesions and an increase in cytokine and immune cell levels in the cerebrospinal fluid, as well as a significant improvement in the patient’s quality of life.

**Table 4:** Top 10 co-cited references in the research on glioma immunotherapy.

Rank	Title	Author	Total Citations	TC per Year	Normalized TC
1	Regression of Glioblastoma after Chimeric Antigen Receptor T-Cell Therapy	BROWN CE	997	124.63	17.11
2	A single dose of peripherally infused EGFRvIII-directed CAR T cells mediates antigen loss and induces adaptive resistance in patients with recurrent glioblastoma	O’ROURKE DM	926	132.29	16.87
3	Neoadjuvant anti-PD-1 immunotherapy promotes a survival benefit with intratumoral and systemic immune responses in recurrent glioblastoma	CLOUGHESY TF	645	129	12
4	Current state of immunotherapy for glioblastoma	LIM M	633	105.5	11.7
5	Management of glioblastoma: State of the art and future directions	TAN AC	619	154.75	22.42
6	Effect of Nivolumab vs Bevacizumab in Patients With Recurrent Glioblastoma The CheckMate 143 Phase 3 Randomized Clinical Trial	REARDON DA	498	124.5	18.04
7	The role of human glioma-infiltrating microglia/macrophages in mediating antitumor immune responses	HUSSAIN SF	435	24.17	6.71
8	Immune and genomic correlates of response to anti-PD-1 immunotherapy in glioblastoma	ZHAO J	415	83	7.72
9	PD-L1 expression and prognostic impact in glioblastoma	NDUOM EK	362	45.25	6.21
10	Anti-Fas/APO-1 antibody-mediated apoptosis of cultured human glioma cells. Induction and modulation of sensitivity by cytokines	WELLER M	360	12	4.46

After normalizing the total number of citations by R software, the highest score (22.42) was achieved by a review from TAN AC et al. published in 2020 in *CA-A CANCER JOURNAL FOR CLINICIANS (* 31 *)*. This article reviewed the current mainstream treatment of glioblastoma. For newly diagnosed tumors, standard treatments included concurrent radiotherapy with temozolomide and further adjuvant temozolomide after surgery. For recurrent tumors, surgery, radiotherapy, chemotherapy or systemic therapy with bevacizumab were all alternative treatments. Finally, the author suggested that in order to improve the prognosis of patients with glioblastoma, more biomarker-based treatment methods should be carried out in the future.

**Figure 7:**
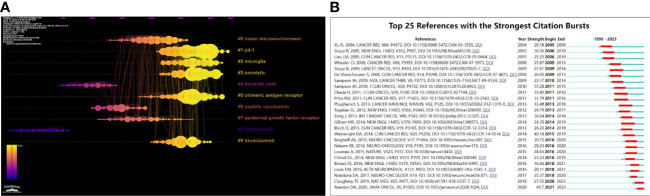
**(A)** Timeline visualization of co-citation clusters contributing to glioma immunotherapy. Each node on the timeline of each cluster represents a reference, and the color of each cluster represents its main time of existence. The size of each node represents the cited times of this reference. **(B)** Top 25 references with the strongest citation bursts. The red segment represents the begin and end year of the burst duration.

The cluster analysis of co-cited references can reveal the knowledge structure in a research field. Clusters were generated by log-likelihood ratio (LLR) algorithm based on the keywords extracted from the co-cited references (32). The top 11 clusters are depicted in **Supplementary Figure 4**. The number of references (size), silhouette value, mean publication year of cited references (i.e., the main year of this cluster), and label of each cluster obtained by LLR algorithm are listed in **Table 5**. The overall modularity and weighed mean silhouette of all the clusters were 0.7676 and 0.9185, respectively, suggesting that the clustering results were convincing. In particular, the silhouette value of each cluster was higher than 0.8, indicating that the co-cited references in one cluster were well matched, with a high heterogeneity. Cluster#0 (tumor microenvironment) was the largest one consisting of 187 co-cited references, followed by Cluster#1(pd-1), Cluster#2 (microglia), Cluster#3 (oncolytic), Cluster#4 (dendritic cells), Cluster#5 (chimeric antigen receptor), Cluster#6 (peptide vaccination), Cluster#7 (epidermal growth factor receptor), Cluster#8 (interleukin-2) and Cluster#9 (bevacizumab). A timeline was used to visualize the references in each cluster and the association between clusters (**Figure 7A**). Cluster#1 had the largest number of periods, while Cluster#8 contained studies that had started earlier. From the perspective of time, Cluster#0, #4, #6-8 all contained early studies with closer connections. From the perspective of label, most of them were early treatment strategies for glioma. However, Cluster#1-3, 5, #9, which demonstrated more and newer references, contained four relatively new therapeutic strategies, namely PD-1, oncolytic therapy (virus therapy), CAR-T therapy and bevacizumab.

**Table 5:** Major clusters of co-cited references contributing to glioma immunotherapy.

Cluster ID	Size	Silhouette	Mean (Year)	LLR
0	187	0.882	2010	tumor microenvironment
1	158	0.875	2015	pd-1
2	142	0.853	2017	microglia
3	126	0.803	2018	oncolytic
4	126	0.969	1999	dendritic cells
5	124	0.908	2016	chimeric antigen receptor
6	104	0.949	2004	peptide vaccination
7	98	0.932	2007	epidermal growth factor receptor
8	83	0.991	1991	interleukin-2
9	61	0.937	2015	bevacizumab

The analysis of co-cited references can outline the trends in a certain research field, and predict the research hotspots in the future (14). Its results become more pronounced if further processed with co-cited reference bursts. Selection criteria of co-cited references using CiteSpace were as follows: the number of states=2; γ [0,1] =1.0; minimum duration=2. The top 25 co-citation bursts are shown in **Figure 7B**. Among them, the first co-citation burst emerged in 2001, when an article was published by *Cancer Research*, titled “Vacation of malignant glioma patients with peptide-pulsed dendritic cells elicits systemic cytotoxicity and internal T-cell infiltration” Yu JS (33). It reported the feasibility, safety, and biological activity of a DCs-based vaccine in the treatment of malignant glioma. It can be seen that when immunotherapy for glioma had just slipped into the research hotspot in the early 21^st^ century, DCs were then exploited to design a breakthrough treatment.

It is worth noting that as of 2023, two articles are still in a prominent state, including “Neoadjuvant anti-PD-1 immunotherapy promotes a survival benefit with intratumoral and systemic immune responses in recurrent glioblastoma” authored by Cloughey TFADDIN and published in *Nature Medicine* in 2019 (34) and “Effect of Nivolumab vs Bevacizumab in Patients With Recurrent Glioblastoma: The CheckMate 143 Phase 3 Randomized Clinical Trial” authored by Reardon DA and published in *JAMA Oncology* in 2020 (35). Cloughey TF et al. found that neoadjuvant therapy of PD-1 blockers was stronger anti-tumor effects on GBM (34), while Reardon DA et al. confirmed through clinical studies that the safety of PD-1 inhibitor Nivolumab in the treatment of GBM was consistent with those in other tumor types (35). With two reports on PD-1 inhibitors in glioma therapy highlighted during this period, it can be tentatively assumed that PD-1 inhibitors remain in the mainstream of glioma immunotherapy research.

### 3.6 Analysis of keywords

**Figure 8:**
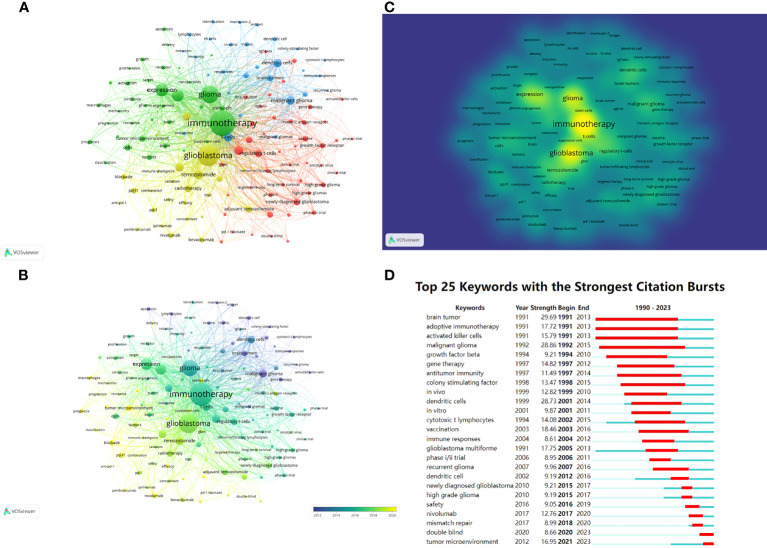
**(A)** Network visualization of keywords based on VOSviewer. All keywords are divided into 4 clusters of 4 colors: green, red, yellow, and blue. **(B)** Overlay visualization of keywords based on VOSviewer. The color of each keyword corresponds to the color of the timeline in the bottom right corner, with purple or blue representing relatively early keywords and yellow representing current hot keywords. **(C)** Density visualization of keywords based on VOSviewer. Keywords with higher weights are brighter. **(D)** Top 25 keywords in the strongest citation bursts. The red segment represents the begin and end year of the burst duration.

Keywords can also be analyzed to display research hotspots and directions. Keywords that had been cited for more than 30 times were visualized and presented in **Figure 8A** through VOSviewer. The center node was immunotherapy (total link strength 7724), followed by glioblastoma (total link strength 5596). **Figure 8B** shows the distribution of keywords over time, and the most recent included “tumor microenvironment”, “prognosis”, “blockade”, “classification”, “pd-l1”, “pembrolizumab” and so on. **Figure 8C** shows the density distribution of keywords. The three most densely cited keywords were immunology (1273 times), glioblastoma (905 times) and glioma (697 times).

CiteSpace was used to detect keyword bursts (**Figure 8D**), aiming to discover changes in hotspots over time. Three keywords “brain tumor”, “adoptive immunotherapy” and “activated killer cells” made the earliest burst in 1991. From 1990 to 2023, the keywords with the highest burst intensity were “malignant glioma” (28.86) and “dendritic cells” (28.73). The keywords that had burst out after 2016 included “safety”, “nivolumab”, “mismatch repair”, “double blind” and “tumor microenvironment”, with the latter two remaining in the current hotspot.

## 4 Discussion

To our knowledge, this is the first bibliometric analysis of glioma immunotherapy-related studies to date, which details the knowledge structure and research hotspots in glioma immunotherapy research during the past 33 years.

As of March 27, 2023, a total of 9505 authors from 1988 institutions in 62 countries/regions had published 1929 articles related to glioma immunotherapy in 502 journals. The number of publications per year and the trends in the numbers of publications over years can be analyzed to offer a comprehensive understanding of research in the field. During the 24-year period from 1990 to 2013, the number of studies and reports related to glioma immunotherapy was not large, while after 2015, the publication volume in this field has grown rapidly, which may be due to the discovery of immune amnesty in the CNS. Prior to 2015, it had long been believed that the CNS lacks lymphatic circuits, and Peter Medawar had reported that implantation of foreign grafts into the brain of rodents does not elicit an immune response, while their implantation into the periphery causes immune rejection (36–38). Their experimental data assumed that the CNS is an immune amnesty zone, which may relate to the limits immune-related studies of glioma. However, in 2015, Louveau et al. verified the presence of functional lymphatic vessels within the dural sinus (10). A powerful inflammation can arouse a strong immune response in the brain (2), suggesting that an active immune surveillance may exist in the CNS (9). The knowledge about immune mechanisms in the CNS has been continuously refreshed since 2015, thus igniting the hope of using immunotherapy to treat glioma. Therefore, related research has abounded as never before.

In the analysis of countries/regions, we found that the top countries publishing the most articles were the US, China, Germany, Japan and Italy, led by the US and China. This may hint at a significant connection between research achievement and the economy of a country, which is consistent with opinions previously reported (39). We also know that all these top five countries have constructed a powerful health care system, which may ensure their academic achievements (40). All the data in the present study suggest that the US is a leader in this research field. The publication volume from China was one-half of that from the US, but only one-fifth in terms of total citations, which indicates that the research in China has a big yield, but its quality needs to be improved. As shown by the timeline, more countries chose to cooperate with the U.S. in 2010, while after that, China became the magnet in this field, which indicates the rising influence of Chinese researchers.

Harvard University, Duke University, Johns Hopkins University and University of California San Francisco ranked high in terms of both total publications, total citations, as well as H-index values, proving that their strength in this research field. All these universities are located in the US, which indirectly confirms the absolute centrality of American research in this field. In addition, UTMD Anderson Cancer Center launched fewer publications, but received the highest number of citations, indicating the high quality and recognition of its research. From the centrality value, University of California Los Angeles demonstrated an increasing profile in the field; meanwhile, the centrality values of the top ten institutions were low, which may be related to the fact that currently no institutions can take a permanent core position in this field. Moreover, domestic cooperation was more popular than international cooperation, suggesting that the latter should also be encouraged.

We further found that the publications involved a wide range of disciplines, as shown by the results presented in the dual-map. IF and JCR division are two important indicators for the quality of a journal. In this field of research, most of the top 10 journals with the largest volume had an IF between 5-15 and a JCR Q1 division. And from the co-cited journals, the majority of journals had an IF over 10 and a JCR Q1 division, and some were famous worldwide. It may suggest that glioma immunotherapy still stands in the research frontier, and is being transformed with theoretical and technological advances. Moreover, despite their fruitful results, there is still room for improvement in related research.

Analysis of journals can guide the study design, journal selection, and article submission of researchers in a given field (17, 41). In terms of publication volume, the top three journals were *JOURNAL OF NEURO-ONCOLOGY* (4.506, Q2), *FRONTIERS IN IMMUNOLOGY* (8.787, Q1) and *FRONTIERS IN ONCOLOGY* (5.738, Q2). Among them, *JOURNAL OF NEURO-ONCOLOGY* is a journal specialized in neuro-oncology, while *FRONTIERS IN IMMUNOLOGY* and *FRONTIERS IN ONCOLOGY* are comprehensive journals in the fields of immunology and oncology, respectively. From the results of the dual-map analysis, publications in the fields of Molecular Medicine, Biology, Genetics were frequently cited by publications in the fields of Molecular Medicine, Biology, Immunology, as well as in the fields of Medicine, Medical, Surgery neighborhoods, indicating the focus paid to either basic or clinical research.

A total of 9505 authors worldwide had been involved in research on glioma immunotherapy. Collaboration mainly occurred between authors in a cluster, and seldom between clusters, which has a correlation with the insufficient international cooperation and communications, suggesting that researchers should strengthen international collaboration. In the number of publications and H-index, the gaps between the top ten authors were not wide, but in total citations and average citations, we were surprised to find that the others lagged far behind Weller M, which may indicate that he has a highest reputation among the top ten researchers. Weller M et al. proposed a reason for the failure of adoptive cellular immunotherapy in malignant glioma: immunosuppressive factors such as transforming growth factor-β (TGF-β) released from glioma cells inhibit T-cell proliferation and thus suppress immune responses. However, they found that in vitro killing mediated by Fas/APO-1 was not inhibited by TGF- β. The resistance of human glioma cells to Fas/APO-1 antibody-mediated apoptosis was mainly associated with a low level of Fas/APO-1 expression, but cytokines IFN-γ and TNF-α enhanced the sensitivity to Fas/APO-1-mediated killing. Therefore, they concluded that a multimodal immunotherapy targeting cytokines and Fas/APO-1 may be designed to fight against glioma (42, 43). In addition, they believed that glioma can be treated by antagonizing TGF-β (44).

### 4.1 Development of glioma immunotherapy

The combination of reference clustering, timeline, and bursts better pictured the evolution of glioma immunotherapy research.

#### 4.1.1 Vaccine therapy

In the early period, supported by basic research, vaccine therapies were spawned, including that based on dendritic cells (DCs). DCs are specialized antigen presenting cells that activate T cells. Both Akasaki Y and Kikuchi T’s teams found that fusion cells (FCs) from DCs with glioma cells can effectively activate anti-tumor immune responses, thus imparting lethality against glioma cells in the lateral abdomen or brain of mice (45, 46). Yamanaka R et al. confirmed that in patients treated with DCs vaccine, no serious adverse effects were observed (47). Also, their patients who had received DCs-based vaccine therapy showed a longer survival than those receiving conventional treatment (48). However, one limitation of this vaccine therapy is that it is favorable for patients with resectable tumors, which restricts its wider application (49).

Another form of vaccine is a peptide prepared by targeting tumor antigens or tumor-related antigens to induce immune response in tumor tissues. Widely used is epidermal growth factor receptor variant III (EGFRvIII), a tumor-specific mutant that mediates tumor proliferation and migration. The high expression of EGFRvIII in tumors, such as glioma, and its silencing in normal tissues makes it an ideal target for immunotherapy (50). However, since EGFRvIII is only expressed in 25-30% of GBMs, this vaccine therapy cannot be taken to counter the resting tumors (49, 51). Furthermore, the expression of EGFRvIII in tumor progression is instable (2). Van den Bent MJ found that EGFRvIII loses its specific expression during amplification in half of the GBMs originally expressing EGFRvIII (52). This instability has also been reported in a study by Felsberg J et al (53).

#### 4.1.2 Viral therapy

Viral therapy works by infesting tumor cells to produce cellular damage. In recent years, viruses have been found able to trigger an anti-tumor immune response. Viral therapy can also shift the tumor microenvironment from immunosuppressive to pro-inflammatory (54). Therefore, it is also considered as a type of immunotherapy. Many replication-competent viruses are now used in viral therapy, such as poliovirus, retrovirus, adenovirus, and herpes simplex virus (HSV) (2). Polioviruses, such as polio-rhinovirus chimera (PVSRIPO), act by binding to receptor CD155, which is widely expressed in solid tumors and their microenvironments. Clinical studies demonstrated higher survival rates at 24 and 36 months in patients receiving PVSRIPO immunotherapy than in historical controls, with no neurotoxicity-related adverse events (55). A typical tumor lysis therapy using retroviruses is Toca-511 (a non-cleavage retrovirus expressing cytosine deaminase), which is specific for cells with division potential, like tumor cells that cannot respond to innate immunity and interferons (56). A clinical study by the Cloughesy TF team showed that Toca-511 triggers a durable complete response, and suggested a positive correlation between a sustained response and an overall survival (57).

#### 4.1.3 CAR-T therapy

In the last decade, Chimeric antigen receptor T cell therapy (CAR-T cell therapy) and Immune-checkpoint inhibitors (ICIs) have been prioritized in designing immunotherapies. CAR-T cells activate anti-tumor immunity by expressing chimeric antigen receptor (CAR) on the surface of autologous or allogeneic T cells, targeting tumor-associated antigens and binding, activating T cells and releasing relevant cytokines without relying on MHC molecules for antigen presentation and co-stimulatory molecules (58). Currently, the intensely studied target antigens in CAR-T cell therapy for GBM include IL-13Ra2, EGFRvIII and Her2 (49). In a clinical study conducted by Brown CE’s team, they administered CAR-T therapy targeting IL-13Ra2 to a patient with recurrent GBM, and observed a size reduction in all lesions and an increase in cytokine and immune cell levels in the cerebrospinal fluid, as well as a significant improvement in the patient’s quality of life (30). O’Rourke DM et al., on the other hand, confirmed the feasibility and safety of CAR-T cell therapy targeting EGFRvIII, without non-tumor toxic reactions or cytokine release syndrome in patients (59). However, after treatment, they found an upregulation of immunosuppressive molecules in the tumor environment and an increase in regulatory T cells (Treg), suggesting that overlooking the effects of CAR-T therapy, tumor tissues can still restore the immunosuppressive environment through adaptive regulation on realize immune escape (2, 59), which limits the application of CAR-T cell therapy. Its efficacy may be impaired by the stress metabolic environment of the tumor, such as hypoxia and nutrient deprivation (58). In conclusion, single CAR-T cell therapies cannot eradicate tumors, and should be combined with anti-TGF-β molecules, anti-IL-6 antibodies and immune checkpoint blocking antibodies (such as PD-1/PD-L1 inhibitors), or IDO inhibitors, or macrophage colony-stimulating factor 1 receptor (CSF1R) inhibitors, or prior conditioning chemotherapy (fludarabine and cyclophosphamide) (2, 58, 60).

#### 4.1.4 ICIs

Through the combination of reference clustering, timeline, and bursts, it is not difficult to find that the frequency of PD-1 is high. According to the reference clustering, PD-1 represents one of the largest and most important clusters. PD-1 is densely studied in latest studies, and the references in these studies are frequently cited. Additionally, the latest reference bursts and most of the top 10 co-cited references are related to PD-1 research. In conclusion, immune-checkpoint inhibitors (ICIs) represented by PD-1, are in current research hotspot and the main component in the immunotherapy for glioma.

In recent years, ICIs, especially those targeting CTLA-4 and PD-1/PD-1, have become the cornerstones in major cancer therapies (61, 62). CTLA-4 inhibits the pathway of T cells by binding to ligand CD80 or CD86 expressed in antigen presenting cells (APCs) (63). The combination of PD-1 with its ligand PD-L1 inhibits the activation, migration, and cell-killing ability of T cells (63, 64). ICIs counteract the inhibitory effect of tumor cells on T-cell activation by blocking the binding of co-inhibitory receptors to their ligands, thus enhancing anti-tumor effects (9). Currently, ICIs, such as ipilimumab, nivolumab and pembrolizumab, have been used in the treatment of solid tumors, including melanoma, non-small cell lung cancer, breast cancer, lymphoma, head and neck cancer, bladder cancer and other malignant tumors (65–67). For glioma, ICIs either alone or in combination with multiple drugs or other therapies have shown encouraging results in preclinical trials. In a study by Fecci PE et al, CTLA-4 blockers can achieve a long-term survival in 80% of treated mice, without causing experimental metaplastic encephalomyelitis (68). Reardon DA et al. found that the combination of anti-CTLA-4 and anti-PD-1 can cure 75% of mice (69). Wainwright DA et al. reported that triple blockade of IDO, CTLA-4 and PD-L1 prolongated the survival of all mice in the experiment (70). And PD-1 blockers combined with local radiotherapy can realize a long-term survival in mice with brain tumors in situ (71). In clinical studies, ICIs have shown evident therapeutic efficacy. A CheckMate 143 Phase III randomized clinical trial compared the efficacy of nivolumab and bevacizumab in the treatment of recurrent GBM. Nivolumab enabled a median survival of 9.8 months, and bevacizumab 10.0 months. The safety of nivolumab in treating GBM patients is consistent with that in other tumor types (35). However, the efficacy of ICIs is not ideal. Reiss SN et al. found in a retrospective study that in treating rGBM with pembrolizumab, PFS was only prolonged in a small fraction of patients, with an OS of 4 months (72). Multiple reasons may explain this unsatisfactory clinical efficacy. For example, compared with other tumors, GBM has a lower mutation rate and a low level of T cell infiltration, which reduces the efficacy of ICIs (73). Also, the blood-brain barrier can discount the efficacy of ICIs (2, 9).

Efforts have been released to improve the efficacy of single ICIs, including combination of ICIs with radiotherapy, chemotherapy, as well as neoadjuvant therapy. Schalper KA et al. reported that neoadjuvant nivolumab increases the expression of chemokine transcripts, immune cell infiltration, and TCR clonal diversity in tumor-infiltrating T lymphocytes (74). Cloughesy TF, on the other hand, reported that if treated with neoadjuvant pembrolizumab, the patients who received continuous adjuvant chemotherapy after surgery had a longer overall survival, compared to those who received adjuvant PD-1 blockade therapy after surgery (34). It is foreseeable that as ICI research progresses, more targets will be raked out for ICIs and more types of ICIs will be synthesized in the future. Then how to combine these ICIs, how to combine ICIs with conventional treatments (radiotherapy, chemotherapy, etc.), or how to standardize ICI treatment protocols will be the key to improving the efficacy of ICIs. In the future, ICI research will focus on the search for targets, as well the optimization of therapeutic strategies.

### 4.2 Research hotspot

The analysis of keywords can tease out the hotspots in the research field, while keyword bursts can demonstrate how research hotspots have changed over time. In the early 1990s, “malignant glioma” and “dendritic cells” rushed into the hotspot, and dendritic cells were then used to design vaccines for glioma treatment, because of their antigen-presenting cell characteristics (46). According to VOSviewer’s analysis, popular keywords that are closer to the present time include “tumor microenvironment”, “prognosis”, “blockade”, “classification”, “pd-l1” and “pembrolizumab”. CiteSpace showed that keywords that had burst after 2016 included “safety”, “nivolumab”, “mismatch repair”, “double blind”, and “tumor microenvironment”, with “double blind” and “tumor microenvironment” still in the swim. Based on the outcome of the two software, it is not difficult to find many keywords that are related to the treatment of PD-1, including “PD-L1”, “pembrolizumab” and “nivolumab”, which again suggests that ICIs such as PD-1 is the mainstream immunotherapy for glioma. “Safety” can suggest that, with multiple immunotherapies showing clinical responses for glioma, the survival and ensure the safety of patients should be well concerned, which is also in line with the fundamental principles of drug development. Meanwhile, “tumor microenvironment” was mentioned by both software, reflecting the importance of tumor microenvironment for glioma treatment. Therefore, current immunotherapy research still aims to suppress the immunosuppressive cells by various methods, and to “blockade” the immune escape of the tumor by various small molecules, such as ICI or antibodies. In short, ICIs such as PD-1 are in the current hotspot of glioma immunotherapy. Through ICIs, the immune escape of glioma may be hindered by modulating the tumor microenvironment, thus allowing various therapies to exert their anti-tumor effects. Therefore, the hotspot of future research on glioma immunotherapy will still be ICIs represented by PD-1. Based on the limited number of ICI targets currently available, it is necessary to explore new and more ICI targets and apply them for clinical treatments. In addition, due to the poor efficacy of a single ICI treatment, it is urgent to conduct research on the combination therapy of multiple ICIs and the combination therapy of ICIs and other therapies to increase the anti-tumor effect. In the future, the research of glioma immunotherapy will no longer be limited to the exploration of certain therapeutic methods. It is advisable for researchers to focus on the comprehensive therapy based on ICIs, with the aim of achieving anti-tumor effect by comprehensively inhibiting the tumor microenvironment, so as to improve the prognosis of patients.

### 4.3 Limitations

There are still several limitations to this study. First of all, all publications pertaining to glioma immunotherapy were searched out of the WoSCC. Although it is one of the most reliable online databases (75), some publications may have been missed. Secondly, only publications in English were collected, which means several potential studies in other languages could be missed. Thirdly, since our study is based on published literature, which might introduce publication bias. Accordingly, we expanded the search scope as much as possible to prevent missing literature in order to reduce publication bias. Fourthly, there may have differences in keywords bursts, clustering analysis of co-citations and institutions contributing to glioma immunotherapy due to the limitations of CiteSpace, VOSviewer and other software.

## 5 Conclusion

In summary, this article reviewed the global trends in the research field of glioma immunotherapy. The results showed that research related to glioma immunotherapy had been widely conducted worldwide and reaped heavy fruits, with the US being the biggest contributor and a powerful leader, and China becoming increasingly active and influential in this area. Emerging research trends have been identified by analyzing keyword bursts and clustering of co-citations. Several immunotherapies are available for glioma, including vaccine therapy, viral lysis therapy, CAR-T therapy, and ICIs. Search for ICI targets and comprehensive ICI treatments has entered and will stay in the research hotspot. This study offers a new insight into the knowledge structure and research prospects of glioma immunotherapy via a qualitative, quantitative and visual method.

## Data availability statement

The raw data supporting the conclusions of this article will be made available by the authors, without undue reservation.

## Author contributions

TZ, YQ, and TC: writing-review and editing. YY, YS, and YW: writing-original draft. YX, YZZ, YYZ and MZ: data curation. All authors contributed to the article and approved the submitted version.
