# Supplementary material for: Knowledge structure and hotspots research of glioma immunotherapy: a bibliometric analysis
Source: Front Oncol. 2023 Aug 21;13:1229905. doi: 10.3389/fonc.2023.1229905 (PMC10476340; doi:10.3389/fonc.2023.1229905)
Supplement: Supplementary file 1 [file DataSheet_1.docx]

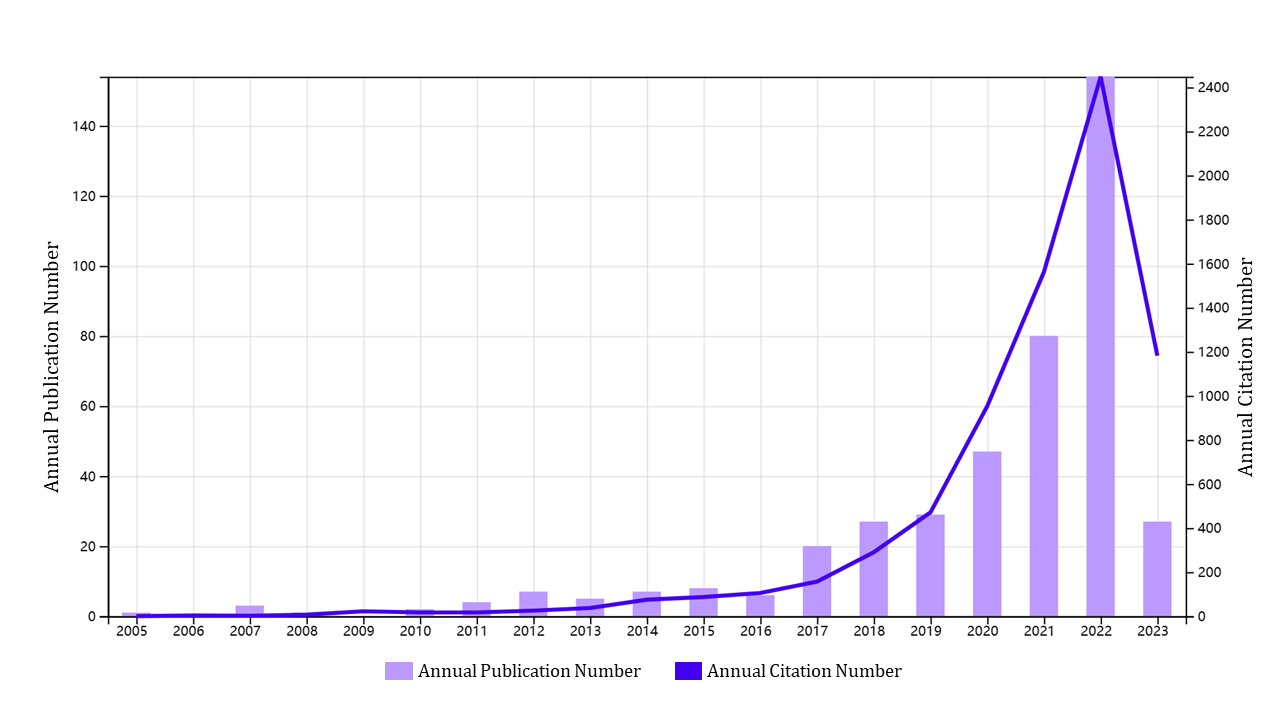


Supplementary Figure S1. A trend in China’s annual publications on glioma immunotherapy. The purple column represents annual publication number, and the blue line represents the development trend of annual citation number.


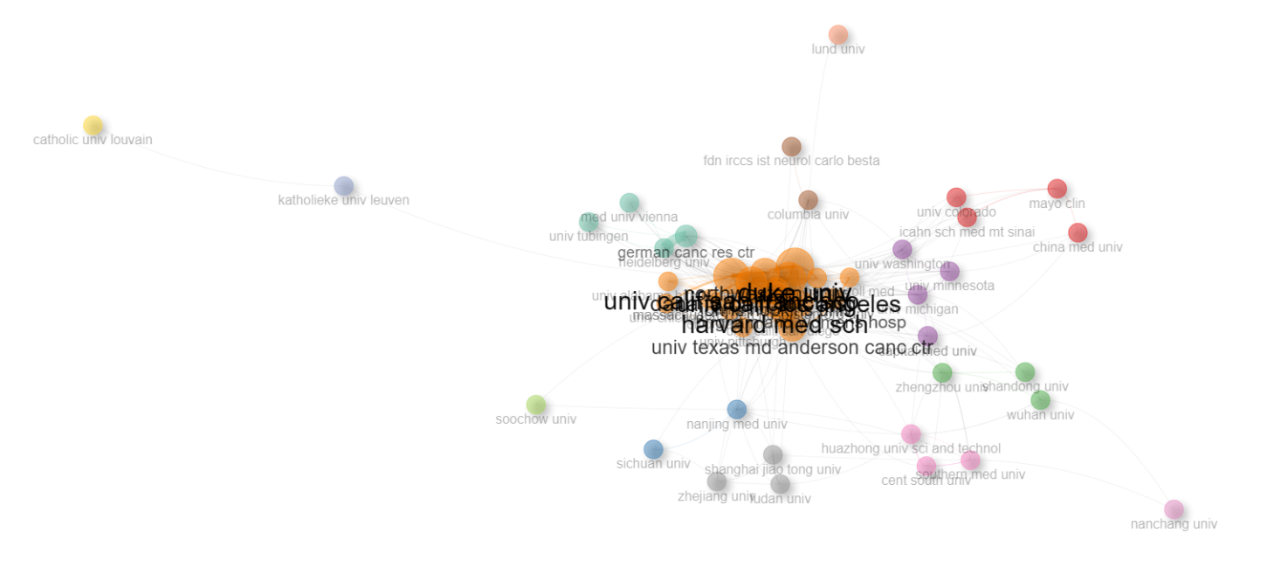


Supplementary Figure S2. The network of institution collaboration contributing to the research on glioma immunotherapy by R software. Each node represents an institution, and the connection line between nodes represents a cooperative relationship between the two. Nodes with the same color represent that they belong to the same cluster, indicating that their research are similar.


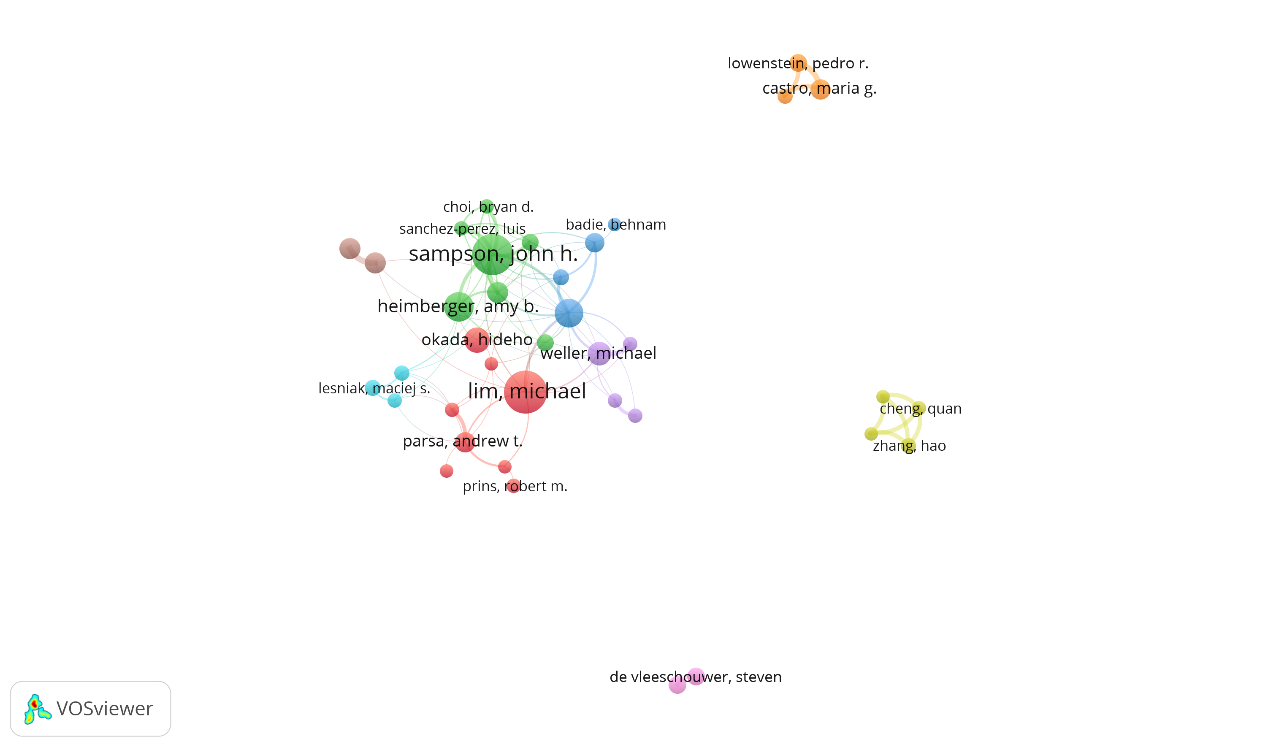


Supplementary Figure S3. A network analysis of collaborations of authors contributing to the research on glioma immunotherapy by VOSviewer. Each node represents an author. The size of the node represents the number of publications issued by the author. Nodes with the same color represent that they belong to the same cluster, indicating that their research are similar.


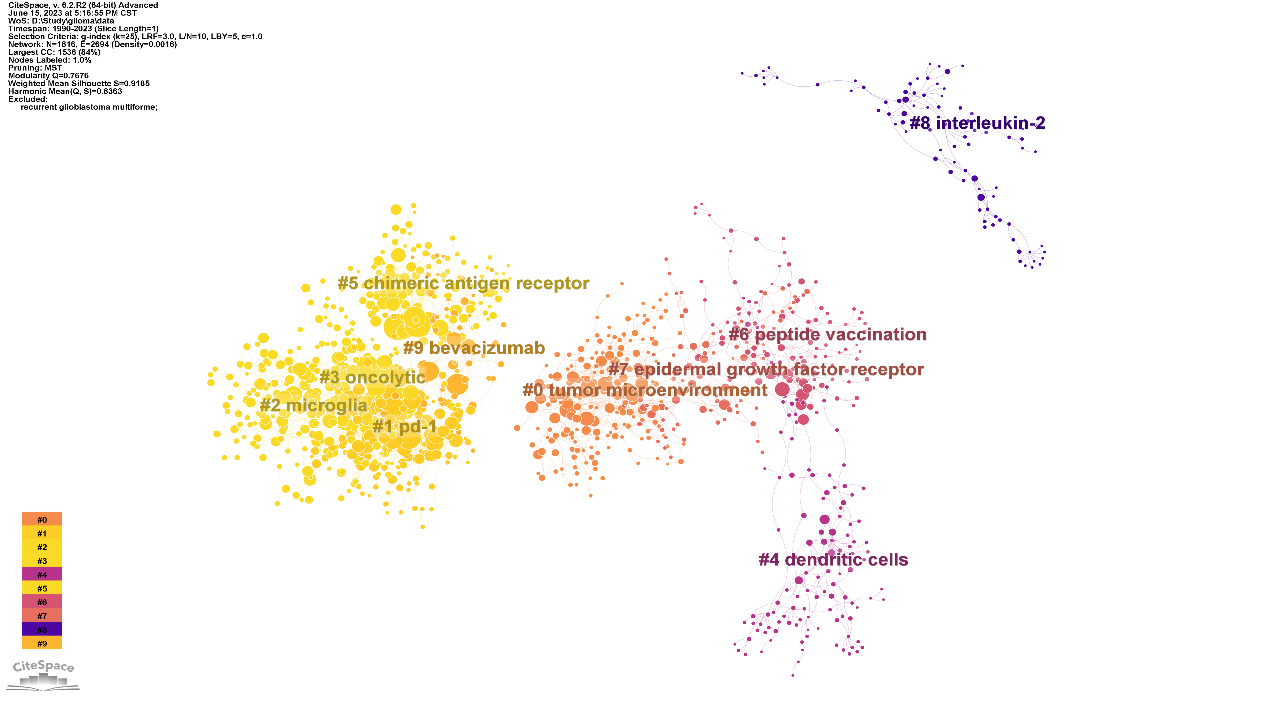


Supplementary Figure S4. A network map showing clusters of co-cited references contributing to glioma immunotherapy.
